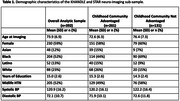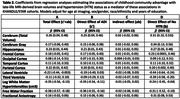# Midlife hypertension status does not mediate associations between childhood community advantage and MRI‐derived markers of late‐life brain health

**DOI:** 10.1002/alz.092760

**Published:** 2025-01-09

**Authors:** Rachel Peterson, Paola Gilsanz, Kristen M. George, Pauline Maillard, Marcia Pescador Jimenez, Lisa L. Barnes, Charles Decarli, Rachel A. Whitmer

**Affiliations:** ^1^ University of Montana, Missoula, MT USA; ^2^ Kaiser Permanente Northern California Division of Research, Oakland, CA USA; ^3^ University of California, Davis, Davis, CA USA; ^4^ Boston University School of Public Health, Boston, MA USA; ^5^ Rush University Medical Center, Chicago, IL USA

## Abstract

**Background:**

Prior research suggests living in an advantaged community in childhood is protective for late‐life cognition and MRI‐derived markers of brain health, independent of individual socioeconomic status. We examined the extent to which this effect is mediated by midlife hypertension status.

**Method:**

KHANDLE and STAR are racially/ethnically diverse cohorts of older adults residing in northern California. A random subset of participants underwent 3T MRI; regional brain volumes and white matter integrity were derived. Participants’ self‐reported birth address was geocoded and linked to historical county‐level Area Deprivation Indices (ADI) for the Census decade of residence (1920‐1970). Childhood community advantage was defined as ADI<20, (i.e., most advantaged quintile nationally). Midlife hypertension was derived from prospectively collected blood pressure (bp) at ages 30‐45 and defined as systolic/diastolic bp ≥130/80 or prior hypertension diagnosis. Generalized linear models estimated the direct (c’) and total effects (ab+c’) of ADI on imaging measures among participants age 65+, adjusting for age, sex, race/ethnicity, and education. Indirect effects (ab) via midlife hypertension were calculated by subtracting direct from total effects. Standard errors were bootstrapped with 500 replications.

**Result:**

Imaging participants (n = 392) from advantaged childhood communities were younger (mean age = 72.6 vs. 76.4), had more education (15.3 vs. 14.3 years) and were less likely to have midlife hypertension (49% vs. 58%; Table 1). The total effect of childhood community advantage was associated with better MRI‐derived measures of brain health (e.g., total gray β = 0.26, 95%CI = 0.04, 0.48; lateral ventricle β = ‐0.22, 95%CI = ‐0.44, ‐0.003; log white matter hyperintensity volumeβ = ‐0.35, 95%CI = ‐0.56, ‐0.13; Table 2, column A). The direct effect of not having midlife hypertension was associated with better MRI‐derived measures of late‐life brain health (e.g., total gray β = 0.31; 95%CI = ‐0.10, 0.53); Table 2, column D). Null indirect effects (Table 2, column C) indicate the effects of childhood community advantage on MRI‐derived measures are not mediated by midlife hypertension status.

**Conclusion:**

Late‐life brain health benefits among participants from an advantaged childhood community were not explained by the absence of midlife hypertension. Future work will investigate additional modifiable risk/protective factors as alternative mechanistic pathways by which community advantage may affect late‐life brain health.